# Exome sequence analysis of rare frequency variants in Late-Onset Alzheimer Disease

**DOI:** 10.1007/s11011-023-01221-7

**Published:** 2023-05-10

**Authors:** Sudharsana Sundarrajan, Arthi Venkatesan, Udhaya Kumar S, Mohanraj Gopikrishnan, Iftikhar Aslam Tayubi, M Aditya, Gowrishankar Bychapur Siddaiah, C. George Priya Doss, Hatem Zayed

**Affiliations:** 1 Independent Researcher, Amsterdam, Netherlands; 2 BIOVIA Specialist, VIAS 3D, MG Road, Bengaluru, 560001 Karnataka India; 3 Laboratory of Integrative Genomics, Department of Integrative Biology, School of BioSciences and Technology, Vellore Institute of Technology, Vellore, Tamil Nadu 632014 India; 4 Department of Computer Science, Faculty of Computing and Information Technology, King Abdulaziz University, Jeddah, Kingdom of Saudi Arabia; 5 Department of Biotechnology, Siddaganga Institute of Technology, Tumkur, Karnataka 572103 India; 6 Department of Biomedical Sciences College of Health Sciences, QU Health, Qatar University, Doha, Qatar

**Keywords:** Alzheimer disease, East-Asian, LOAD, Exome, Rare variants, Molecular Dynamics simulation

## Abstract

Alzheimer disease (AD) is a leading cause of dementia in elderly patients who continue to live between 3 and 11 years of diagnosis. A steep rise in AD incidents is observed in the elderly population in East-Asian countries. The disease progresses through several changes, including memory loss, behavioural issues, and cognitive impairment. The etiology of AD is hard to determine because of its complex nature. The whole exome sequences of late-onset AD (LOAD) patients of Korean origin are investigated to identify rare genetic variants that may influence the complex disorder. Computational annotation was performed to assess the function of candidate variants in LOAD. The in silico pathogenicity prediction tools such as SIFT, Polyphen-2, Mutation Taster, CADD, LRT, PROVEAN, DANN, VEST3, fathmm-MKL, GERP +  + , SiPhy, phastCons, and phyloP identified around 17 genes harbouring deleterious variants. The variants in the *ALDH3A2 and RAD54B* genes were pathogenic, while in 15 other genes were predicted to be variants of unknown significance. These variants can be potential risk candidates contributing to AD. In silico computational techniques such as molecular docking, molecular dynamic simulation and steered molecular dynamics were carried out to understand the structural insights of RAD54B with ATP. The simulation of mutant (T459N) RAD54B with ATP revealed reduced binding strength of ATP at its binding site. In addition, lower binding free energy was observed when compared to the wild-type RAD54B. Our study shows that the identified uncommon variants are linked to AD and could be probable predisposing genetic factors of LOAD.

## Introduction

Alzheimer disease (AD) is a chronic, progressive neurodegenerative disorder. AD is characterized by the accumulation of extracellular neurotic beta-amyloid plaques and intracellular neurofibrillary tangles composed of hyper-phosphorylated Tau protein leading to neuronal death and cerebral atrophy (Ballard et al. 2011; Harris 2012; Holtzman et al. 2011). The disorder is associated with cognitive dysfunctions and psychiatric and behavioural disturbances. AD significantly contributes to dementia, affecting around 35 million people worldwide. Based on the onset age, AD is classified as early-onset (age < 65 years) and late-onset (age >  = 65 years). Around 10% of AD patients are diagnosed with early-onset AD (EOAD) (Bekris et al. 2010). Studies have documented that the incidence rates for familial dementia and LOAD are significantly higher than population-based estimates. LOAD encompasses complex aetiology with a heritability rate of 58–79% (Vardarajan et al. 2014; Awada 2015).

The prevalence and incidence of AD indicated age as the most influential known risk factor. Early efforts in understanding AD were mainly focused on EOAD. These studies were centred on large multi-generation families' harbouring clear autosomal dominant patterns of inheritance related to mutations in genes that alter amyloid-beta (Aβ) protein production, aggregation, or clearance. EOAD patients were often associated with 3 common genes; Amyloid Beta Precursor Protein (*APP)* and Presenilin genes (*PS1* and *PS2*), which occur in half of the EOAD cases. The genetic basis of LOAD is more complex, with susceptibility likely conferred by various common but less penetrant genetic factors, such as apolipoprotein E (APOE) alleles, interacting with environmental and epigenetic factors. Although substantial evidence indicates genetic factors as a key player, APOE-4, the only identified LOAD gene seems to act as a primary modifier at the age of onset and in patients with onset before age 70 years (Blacker and Tanzi 1998) (Bekris et al. 2010) (Bellenguez et al. 2019). The APOE-4 allele was overrepresented in both AD divisions. APOE-4/4 homozygotes act as a risk factor in LOAD cases.

Apart from complex genetics, other risk factors contribute to the difficulty in identifying LOAD genes. i) The base rate of LOAD cases is high and increases steeply with age. Hence clustering among families can occur due to chance alone, and several sources of the disease may co-occur in the same family. ii) LOAD occurs at the end of life span, and many individuals do not survive to the age of risk. iii) Elderly patients are prone to other sources of cognitive decline, diluting the power of genetic studies with individuals carrying the disease but are not actual gene carriers (phenocopies) (Blacker and Tanzi 1998) (Andrade-Guerrero et al. 2023). An early diagnosis of the disease is highly crucial for effective treatment. An early diagnosis helps the affected individuals to explore and benefit from drug and non-drug treatment available. An early diagnosis opens prospects for participating in wide range of clinical trials, leading to advancing research and providing medical benefits. The current treatment strategies mainly focus on reliving and delaying the progression of the symptoms.

Large-scale genome-wide association studies (GWAS) and meta-analyses of the GWAS have identified more than 30 different LOAD-susceptible loci, focusing on the European population (Karch and Goate 2015). These GWAS hits documented polymorphisms, mostly in intronic and intergenic regions. In addition, whole-exome microarray and whole-exome sequencing (WES) also contributed to identifying rare and novel variants. Recently, whole exome sequencing techniques have been utilized to understand the mutational mechanisms in several diseases, including cancer (Kumar et al. 2022). These advanced techniques also help to comprehend the structural mechanisms disrupted upon mutations using computer simulations and docking methods (Udhaya Kumar et al. 2022) (Kumar and Priya Doss 2021) (Tayubi et al. 2022).

The genetic diversity existing among different ethnic groups may have an effect on genetic factors in the pathogenesis of AD. AD genetic and clinical sucecptibility profile seems to be different with different ethnic groups (Al-Thani et al. 2021). Most of the genetic studies on AD were based on European cohorts, implying the absence of ethnic diversity data in genetic research. Including genetic studies on other populations, including East Asians, can lead to exhaustive genetic details of AD pathogenesis (Miyashita et al., 2022). The current work utilizes the distinct genetic profiles of Koreans with AD to discover LOAD-associated genes and variants. Higher AD occurrences are observed among older Koreans in East-Asian populations (Jang et al. 2021). Whole-exome sequencing analysis of post-mortem hippocampal regions from AD patients and age-matched healthy controls of Korean ethnicity is performed to identify novel LOAD risk genes.

## Materials and methods

### Data retrieval

The exome sequences of LOAD-affected individuals and control samples were retrieved from NCBI SRA (Accession: PRJNA532465) (Leinonen et al. 2011). Post-mortem hippocampal regions of 52 AD and 11 age-matched control samples were retrieved. About 37 LOAD samples were selected based on the disease onset (age ≥ 65 years). 15 samples were from EOAD cases (age < 65 years) and used to verify rare variants. The disease stage, ethnicity, and gender were also verified for the samples (Table 1). The selected samples were chosen for further analysis.

**Table 1:** Baseline characteristics of study subjects

Category	LOAD	EOAD	Control
No. of individuals	37	15	11
Gender	12 M; 25 F	4 M; 11 F	7 M; 4 F
Age, mean ± SD (range)	77.16 ± 7.16	61.06 ± 21.3	74.09 ± 9.43
Onset age, mean ± SD	84.7 ± 86.06	54.75 ± 5.41	-
Stage – High (4–6)	32	13	0 (0/11)
Stage – Low (0–3)	4	2	100% (11/11)

### Data processing

The quality control of the raw paired-end exome sequences was carried out using FastQC. Following quality control, high-quality reads were mapped to human genome build GRCh38 using Burrows-Wheeler Aligner (Li and Durbin 2009). The mapped reads were duplicate marked, and base quality score recalibration was carried out using Picard and GATK suite. GATK suite was employed to identify variants. The variants were annotated using Annotate Variation (ANNOVAR) tool (Wang et al. 2010). Non-synonymous exonic variants with Minor Allele Frequencies (MAF < 0.01) were prioritized to identify rare variants. Only coding variants were selected. Variant pathogenicity was assessed using SIFT, Polyphen-2, Mutation Taster, CADD, LRT, PROVEAN, DANN, VEST3, fathmm-MKL, GERP, SiPhy, phastCons, and phyloP tools (Doss and Zayed 2017; Liu et al. 2020). The final candidate variants were prioritized by utilizing UKBiobank PheWeb, GeneCards (Safran et al. 2010), OMIM (Amberger et al. 2015), and ACMG guidelines (Harrison et al. 2019).

### Docking

Using the molecular docking approach, users can theoretically screen a library of chemicals and forecast the most potent binding sites using a variety of scoring algorithm (Agrahari et al. 2018a, 2019; Ali et al. 2017). Using Autodock Vina docking software, the docking analysis of the ligand ATP with RAD54B was performed. The predicted active site was the primary target for docking the protein–ligand complex. The anticipated active site of RAD54B was then docked with arylidenes ATP using AutodockVina (Trott and Olson 2010). The pose with the highest AutodockVina dock score was then chosen for molecular dynamics simulation studies.

### Protein–ligand dynamic simulation (MDS)

Selected Protein–Ligand complexes from docking data were subjected to MDS using Gromacs-2019 (Agrahari et al. 2018b; Abraham et al. 2015). We obtained the chosen ligand topology by downloading it from the PRODRG website. Utilizing the steepest descent technique, we setup the system and reduced the vacuum for 1500 steps (Petrova and Solov'ev 1997). The structures in a cubic periodic box of 0.5 nm were solvated using the simple point charge (SPC) water model. It was, therefore, sufficient to sustain the complex system with a salt concentration of 0.15 M by introducing an adequate amount of Na^+^ and Cl^−^ counter ions. The system setup was covered based on a previously published work. The ensemble underwent a final simulation run of 100 ns following the NPT equilibration stage. The trajectory was examined using various GROMACS analytic techniques as a last step. The RMSD, RMSF, Rg, solvent accessible surface area (SASA), and intramolecular H-bonds between our protein molecules were calculated for the wild and all mutant proteins, respectively, using the gmx rms, gmx rmsf, gmx gyrate, gmx sasa, and gmx hbond. Molecular Mechanics Poisson-Boltzmann Surface Area (MM-PBSA) was employed to comprehend a substrate's binding free energy (ΔG binding) with a protein throughout the simulation. The GROMACS function g_mmpbsa was utilized to estimate the ΔG binding. Within the final 1000 frames, in the last 50 ns, computation ΔG produced our results (Homeyer and Gohlke 2012).

### Steered molecular dynamics

We ran a brief MD run on each system to establish equilibrated structures before running the SMD simulation (Izrailev et al. 1999). The Alphafold structure of RAD54B uploaded to UniProt served as the basis for the MD simulations. The GROMOS54A7 force field was used to run the simulations for both the wild and mutant RAD54B-ATP systems (Huang et al. 2011). GROMOS54A7 force field compatible parameters for the ATP molecule were retrieved from the PRODRG server (Schüttelkopf and van Aalten 2004). The structures were subjected to vacuum energy minimization before using the steepest descent algorithm. With the help of the SPC model, water molecules extended 20 Å from the protein across all sides of the cubic box in which the structures were solvated. Using the steepest descent algorithm, an appropriate number of ions was added to maintain a salt concentration of 0.15 M. The systems were energy-minimized for 5000 steps. The systems were first brought into equilibrium in the NVT ensemble and then in the NPT ensemble for 100 ps each. A V-rescale thermostat 37 with a coupling constant of 0.1 ps was used to maintain the physiological temperature of 310 K, and a Parinello-Rahman barostat with isotropic pressure coupling was used to maintain the pressure at 1 bar. The long-range electrostatic interactions were handled by the particle mesh Ewald (PME) sum with a cutoff of 1.0 nm (Essmann et al. 1995). Periodic boundary conditions and a threshold radius of 1.0 nm were employed for van der Waals interactions.

Using the LINCS algorithm, all hydrogen atom bonds were restricted (Hess et al. 1997). The final equilibrated structure from the NPT equilibration step was considered the initial structure for performing Steered Molecular Dynamics (SMD) Simulation Studies. Various biomolecular systems have been evaluated using constant-velocity SMD simulation, and encouraging correlations with experimental results are reported. To investigate the ATP molecule's binding affinity at the ATP binding site, separate MD simulations were run on each system. Using constant-velocity SMD simulations, ATP was pulled from the ATP binding site in each system. The centre of mass of the steered group, or the ATP tail region, and the centre of mass of the protein residues constituting the ATP binding pocket were used to define the pulling vector. The constant velocity simulations used a force constant of 1000 kJ mol-1 nm-2. ATP was drawn with a 5 nm/ns pulling velocity for each system. We emphasize that the systems are frequently severely perturbed when greater pulling rates are used in SMD, and some details are less likely to be recorded at such higher pulling rates. The increased pulling rates can also impair the protein's normal elastic response. The pulling velocity was selected to achieve the ideal balance of precision and computing speed.

## Results and discussion

The exomes of individuals affected with LOAD were compared with healthy controls. Around 85,000 variants per sample qualifying the initial quality control filters were investigated further. The filtering of variants for synonymous variants and known LOAD risk genes (*ABI3, ABCA7, ADAM17, AKAP9, IGHG3, BIN1, CASS4, CD33, CD2AP, CELF1, CLU, CR1, DSG2, EPHA1, FERMUTANT2, HLA-DRB5-DBR1, INPP5D, MS4A, MEF2C, NME8, PICALM, PLD3, PLCG2, PTK2B, SLC24H4-RIN3, SORL1, UNC5C,* and *ZCWPW1*) resulted in the identification of 1531 variants.

### Rare variants identification in LOAD samples

Filtering variants with MAF < 0.01 resulted in 1151 hits. Variants from 21 genes *GJA8, CHRNB3, PGK2, TGM2, GDF9, CCR10, ALDH3A2, CLDN3, GK2, GSR, MUTANTHFS; ST20-MUTANTHFS, CHRNA1, RAD54B, GDA, TMLHE, MIXL1, CPXM1, WNT10A, KLC4, WFDC2,* and *MAGI1* were predicted to be damaging to the protein by SIFT, Polyphen-2, Mutation Taster, CADD, LRT, PROVEAN, DANN, VEST3, fathmm-MKL, and SiPhy. These variants were also predicted to be conserved by GERP, phastCons, and phyloP. Systems-level investigation of these variants was carried out using OMIM and ACMG guidelines. Significant associations were observed between 9 genes (*GJA8, CHRNB3, GDF9, ALDH3A2, GSR, CHRNA1, GDA, KLC4,* and *MAGI1*) and the disease phenotype using UKBiobank PheWeb. The presence of these variants was checked in control and EOAD samples. The comparison showed that variants from 17 genes were absent in control or EOAD cases (Tables 2 & 3). The genes *ALDH3A2* and *RAD54B* harboured likely pathogenic variants, and the remaining 15 genes contained variants of unknown significance. The genes predicted to be deleterious were further assessed for their involvement in the disease.

**Table 2:** Frequency of rare variants in population frequency databases

Gene	1000G_ALL	1000G_EAS	ExAC_Freq	ExAC_EAS	gnomAD_exome_ALL	gnomAD_exome_EAS
*CHRNB3*	0.0004	-	0.0004	0	0.0003	0
*GDF9*	0.0028	-	0.0029	0	0.0030	0
*CCR10*	-	-	0.02122	0	0.004292	0
*ALDH3A2*	-	-	0.0002	0	0.0001	0
*CLDN3*	1	-	0.0045	0	0.0044	0
*GK2*	-	-	0.04942	0	0.007715	0
*GSR*	-	-	0.0001	0	0.0001	0
*MTHFS;ST20-MTHFS*	-	-	0.03295	0	0.03295	0
*RAD54B*	-	-	0.01661	0	0.01661	0
*GDA*	0.0078	-	0.0130	0	0.01310	0
*TMLHE*	-	-	0.0002	0	0.0002	0
*MIXL1*	-	-	0.008284	0	0.08284	0
*CPXM1*	0.0006	-	0.0021	0	0.0021	0
*WNT10A*	-	-	0.03297	0	0.03297	0
*KLC4*	-	-	0.008368	0	0.008368	0
*WFDC2*	-	-	0.0001	0	0.0001	0
*MAGI1*	0.0002	-	0.04123	0	0.02867	0

**Table 3:** Pathogenicity prediction scores of rare variants

Gene	Variant	rsID	SIFT	PolyPhen2 HDIV	Polyphen2 HVAR	LRT	Mutation Taster	DANN	CADD	PROVEAN	VEST3	fathmm- MKL	GERP + +	SiPhy	phast Cons100way vertebrates	phyloP100way_ vertebrate	UKBiobank PheWeb
*CHRNB3*	c.C985T (p.H329Y) c.C763T (p.H255Y)	rs149775276*	0	0.999	0.998	0	1	0.998	5.14	-5.67	0.83	0.96	5.85	20.22	1	5.41	2.50e-09
*GDF9*	c.C1360T (p.R454C) c.C1096T (p.R366C)	rs61754582*	0	1.0	0	0	1	0.999	7.2 2	-7.05	0.89	0.99	5.24	17.1	1	7.68	3.7e-8
*CCR10*	c.G413A (p.R138H)	rs773778989*	0	1.0	0.999	0	1	0.999	7.16	-4.97	0.86	0.98	4.1	15.26	1	7.8	-
*ALDH3A2*	c.C943T (p.P315S)	rs72547571^&^	0	1.0	0.998	0	1	0.935	6.6	-7.82	0.94	0.95	5.12	17.58	1	7.83	2.5e-09
*CLDN3*	c.C401T (p.P134L)	rs139191328*	0	1.0	0.999	0	1	0.999	6.87	-9.39	0.98	0.98	4.94	15.67	1	9.94	-
*GK2*	c.G1318C (p.D440H)	rs202223457*	0	1.0	0.999	0	1	0.994	4.75	-6.16	0.94	0.98	4.21	14.87	1	3.86	-
*GSR*	c.C1253T (p.T418M) c.C1412T (p.T471M) c.C1340T (p.T447M) c.C1499T (p.T500M)	rs200685394*	0	1.0	0.999	0	1	0.999	6.96	-5.8	0.83	0.96	5.34	16.9	1	5.08	2.90e-15
*MTHFS;ST20-MTHFS*	c.G263A (p.R88Q) c.G434A (p.R145Q) c.G362A (p.R121Q)	rs753635972*	0	1.0	1.0	0	1	1	8.22	-3.79	0.94	0.98	6.17	20.88	1	7.19	-
*RAD54B*	c.C824A (p.T275N) c.C1376A (p.T459N)	rs576696900^&^	0	1.0	0.999	0	1	0.994	5.54	-4.16	0.93	1	4.84	14.54	1	10	-
*GDA*	c.G790A (p.V264M) c.G568A (p.V190M) c.G664A (p.V222M) c.G790A (p.V264M) c.G568A (p.V190M)	rs61752956*	0	0.998	0.983	0	0.95	0.999	5.01	-2.56	1	0.83	4.0	6.31	1	3.9	1.8e-13
*TMLHE*	c.T866C (p.F289S)	rs141804314*	0	1.0	0.984	0	1000	0.999	5.91	-6.13	0.86	0.93	4.42	11.02	1	5.03	-
*MIXL1*	c.C436T (p.R146C) c.C412T (p.R138C)	rs766606088*	0	1.0	1.0	0	1000	0.999	7.48	-8.0	0.92	0.95	5.4	15.9	0.99	5.21	-
*CPXM1*	c.G749A (p.R250H)	rs41301840*	0	1.0	999	0	1	1	7.42	-3.49	0.95	0.97	4.66	15.08	1	5.48	-
*WNT10A*	c.G391A (p.A131T)	rs372993798*		1.0	999	0	1	0.999	7.21	-3.25	0.97	0.98	4.7	16.73	1	8.01	-
*KLC4*	c.G856C (p.A286P) c.G1087C (p.A363P) c.G1141C (p.A381P)	rs754560631*	0	1.0	999	0	1	0.998	6.7	-4.77	0.98	0.99	6.05	20.61	1	9.88	3.50e-11
*WFDC2*	c.T145C (p.C49R)	rs368665218*	0	1.0	997	0	1	0.987	4.65	-9.6	0.96	0.52	4.58	10.36	0.99	4.81	-
*MAGI1*	c.C968T (p.T323M)	rs554948124*	0	1.0	996	0	1	0.999	6.86	-5.29	0.76	1	5.89	20.23	1	9.22	3.10e-22

^&^Likely pathogenic, *Variant of unknown significance

### Functional insight into genes associated with LOAD

#### Pathogenic variants

The variants from two genes, *ALDH3A2* and *RAD54B,* were predicted to be deleterious and pathogenic by the in silico tools.

*ALDH3A2* reported two single nucleotide variants (SNV) at exon 7. The gene product is an NAD + oxidoreductase enzyme complex component responsible for oxidizing fatty alcohol to a fatty acid. Mouse knockout studies of the gene showed several abnormalities corresponding to behavioural traits correlating with movement instability and anxiety issues observed in AD patients. The SNV observed at exon 7 of the *ALDH3A2* gene in our sample correlates with the point mutation associated with Sjogren-Larsson Syndrome (SLS). The mutation causes C to T exchange at the nucleotide position 943 in the cDNA leading to the replacement of highly conserved proline to serine (De Laurenzi et al. 1997; Kanetake et al. 2019).

*RAD54B* reported two SNVs at exon 6 and exon 8. The gene is a member of the helicase superfamily involved in recombination and DNA repair. DNA damage is one of the critical pathological causes of AD, as DNA damage accumulation is noted in patients' brains. Defects in DNA damage and repair enzymes such as *RAD54B* may facilitate the disease pathogenesis (Murzik et al. 2008; Lin et al. 2020).

#### Uncertain significant variants

Around 15 genes (*CHRNB3, GDF9, CCR10, CLDN3, GK2, GSR, MUTANTHFS; ST20-MUTANTHFS, GDA, TMLHE, MIXL1, CPXM1, WNT10A, KLC4, WFDC2,* and *MAGI1*) with uncertain significance variants and severe consequences were obtained (Tables 4 and 5).

**Table 4:** The calculated parameters (average values) for all the systems based on 100-ns MD simulation

S. No	Protein	RMSD (nm)	RMSF (nm)	Rg (nm)	SASA (nm^2^)
1	WT-ATP	0.2554	0.1370	2.9687	360.2330
2	MT-ATP	0.2802	0.1582	2.9369	359.2357

*CHRNB3* reported two SNVs in exons 5 and 6. The gene codes for the neuronal nicotinic acetylcholine receptor (nAChR) component. The variant associated with *CHRNB3* may potentially affect the regulation of nAChRs leading to the disruption of transmitter release, neuronal integration, and cell excitability, as documented in many neurological disorders (Hogg et al. 2003; Abu-Amero et al. 2015). *CCR10* reported an SNV in exon 2. *CCR10* is a chemokine receptor expressed in astrocytes. Mutation in the gene may alter its ligand binding, inducing immune cascade disturbances in the Central Nervous System (Liu et al. 2014). *CLDN3* also reported an SNV in exon 1. The gene codes for a component of tight junction strands. *CLDN3* is known to localize in the brain endothelial cells. Any genetic changes in the gene may introduce a breakdown in the blood–brain barrier associated with the endothelial cells (Romanitan et al. 2010). *GDA/Cypin* reported around seven SNV and observed among four samples in our dataset. The gene is localized in dendrites, increasing branching and promoting microtubule assembly. Patients with AD show fewer branches of neurons within the hippocampus, potentially reflecting the loss of learning and memory (Arikkath 2012). *TMLHE* reported two SNVs in exon 6. The gene product is the first enzyme in the carnitine biosynthesis pathway. Carnitine contributes to neuroprotective, neuromodulatory, and neurotrophic functions. Mutations in the gene may affect the carnitine pathway. Loss of function of *TMLHE* is also shown to be associated with autism spectrum disorders (Nałecz et al. 2004; Virmani and Binienda 2004; Nava et al. 2012).

*KLC4* reported five SNVs in exons 7, 8, and 9. Studies on mutant *KLC4* zebrafish revealed that it is crucial for peripheral sensory axon branching and proper arborization. The study also showed altered microtubule dynamics. An increased anxiety-like behaviour was also observed, indicating the role of *KLC4* in neural circuits (Haynes et al. 2022). *MAGI1* reported an SNV in exon 6. The gene plays a significant role in the organization of membrane proteins and cytoskeletons by transmitting signals pertaining to cell–cell or cell–matrix interactions. Mutations in *MAGI1* may result in the perturbation of these cell–cell signalling (Hammad et al. 2016). *WNT10A* reported an SNV in exon 3. *WNT10A*^*−/−*^ knockout mice exhibited spatial memory impairment and anxiety-like behaviour (Zhang et al. 2022). *WNT10A* deficiency was proven to cause hippocampal neuro-degeneration in mice indicating similar effects in AD patients*. WFDC2* reported an SNV in exon 2. Human epididymis protein 4 (HE4), encoded by the *WFDC2* gene, is a secretory protein expressed in human epididymis and an important biomarker for ovarian epithelial cancer (James et al. 2018). Studies on serum levels of HE4 revealed it as a sensitive biomarker for the early recognition of the cognitive decline in patients suffering from diabetes mellitus (Bai et al. 2020). As observed in AD patients, impairment in the WFDC2 gene function may lead to cognitive decline.

One of the limitations of the current study is that no literature support was found to assess the function and involvement of other genes (*GDF9, GK2, GSR, MUTANTHFS; ST20-MUTANTHFS, MIXL1 and CPXM1*) in AD or other neurological disorders.

#### Molecular dynamics (MDS)

Through 100-ns MD simulations, we carried out all-atom MDS to examine the consequences of the complex mutation T459N on the structural integrity of the RAD54B protein [Wild type with ATP (Wild-ATP) and mutant type [T459N] with ATP (Mutant-ATP)].

**Fig. 1:**
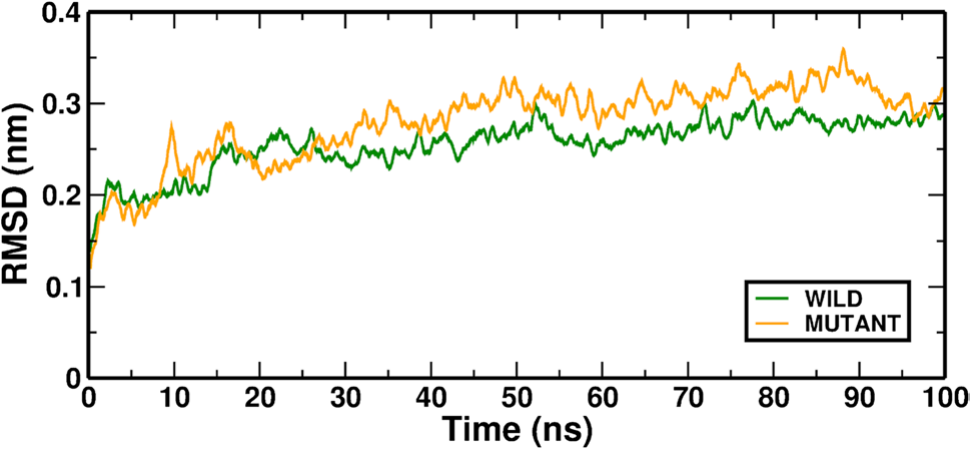
In MD simulation, the RMSD value is averaged over the Wild-ATP and Mutant-ATP complexes

After 20 ns of observation, we discovered that these trajectory motions grew steadier. As a result, the second half of the trajectory was considered for additional investigation. The mutant complex's RMSD measurements did not reveal appreciable variations in these outcomes. The RMSD of Wild-AT and Mutant-ATP complex is illustrated in (Fig. 1). The RMSD value has produced a consistent trajectory, offering a good foundation for further research. The Wild-ATP, Mutant-ATP, and average RMSD values were 0.2554 and 0.2802.

**Fig. 2:**
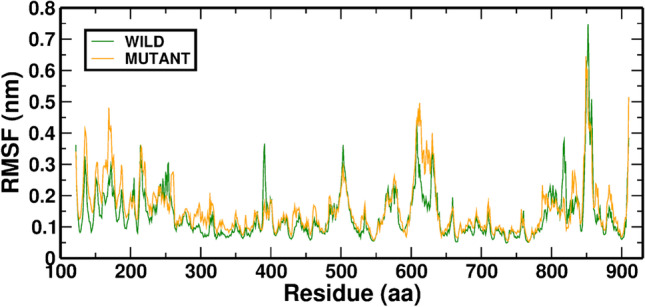
In MD simulation, the RMSF value is averaged over the Wild-ATP (RAD54B) and Mutant-ATP complexes

To evaluate and fully grasp the effects of the Wild-AT and Mutant-ATP complex on the flexible areas of the RAD54B, the Ca residue (RMSF) was determined from its time-averaged stance. The RMSF describes that the residue backbone adopts a higher level of fluctuation in Mutant-ATP than in the Wild-ATP complex. It is hypothesized that the mutation T459N alters how ATP binds to the protein and increases the backbone's flexibility. The RMSF information of Wild-ATP and Mutant-ATP complexes is illustrated in (Fig. 2).

**Fig. 3:**
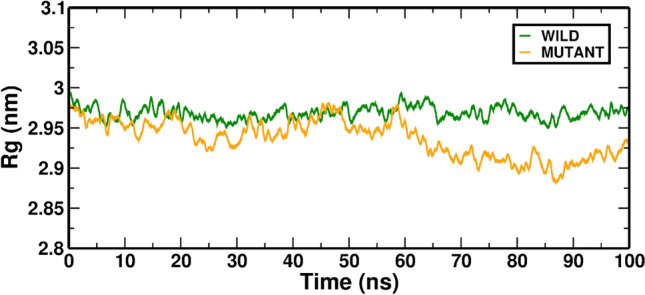
In MD simulation, the RG value is averaged over the Wild-ATP (RAD54B) and Mutant-ATP complexes

The mass-weighted root mean square distance of atoms from their centers of mass can be used to characterize the radius of gyration (Lobanov et al. 2008). The Rg figure shows the competency and form folding of the entire RAD54B structure at various times during the trajectory (Fig. 3). Throughout the simulation, the Wild-ATP complex exhibited a nearly similar Rg value of which the Mutant-ATP complex showed a higher deviation of Rg. As a result, the mutant protein has become more compact, resulting in a slower folding rate than the Wild-ATP.

**Fig. 4:**
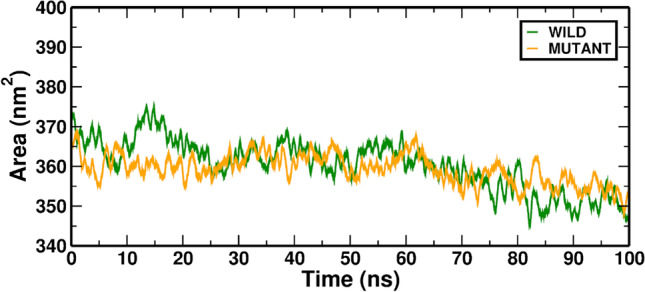
In MD simulation, the SASA value is averaged over the Wild-ATP (RAD54B) and Mutant-ATP complexes

To assess the hydrophobic core's compactness, the SASA change was studied. The change of SASA of the Wild-ATP and Mutant-ATP complexes with time is shown in (Fig. 4). Both complexes exhibited a nearly similar SASA value throughout the simulation.

**Fig. 5:**
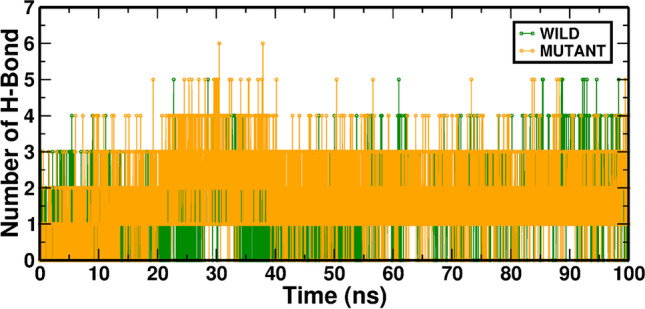
In MD simulation, the H-bond interaction of Wild-ATP (RAD54B) and Mutant-ATP complexes

The H-bonds numbers formed between RAD54B and ATP during the MDS were also evaluated. The H-bond profile was varied, fluctuating from 0 to 6 with a median of 3 H-bonds in the Wild-ATP and Mutant-ATP complexes (Fig. 5). The average values for the WT-ATP and MT-ATP simulations are provided in Table 5.

**Table 5:** Active binding pocket molecular mechanics Poisson-Boltzmann Surface Area (MM-PBSA)

WT-ATP		MT-ATP	
Residue (aa)	Binding Energy (kcal/mol)	Residue (aa)	Binding Energy (kcal/mol)
GLU-432	2.761	ILE-323	-0.078
HIS-434	0.156	GLU-432	1.86
ARG-435	-3.473	GLY-433	-0.166
LEU-436	-0.614	HIS-434	-2.159
LYS-437	7.822	ARG-435	0.263
THR-459	-0.621	LEU-436	-0.236
GLY-460	-0.616	LYS-437	1.949
THR-461	-1.862	ASN-438	-0.02
PRO-462	-4.95	ASN-459	-0.143
ILE-463	-5.679	GLY-460	-0.109
GLN-464	-5.244	THR-461	-0.543
ASN-465	-0.33	PRO-462	-0.334
ASP-466	-0.295	ILE-463	-1.162
GLU-469	-1.022	GLN-464	-0.042
ASN-668	-0.059	ASN-465	-0.583
SER-719	-0.088	ASP-466	-0.228
SER-720	0.154	LEU-467	-0.079
LYS-721	-1.15	GLN-468	-0.15
ALA-722	-0.19	GLU-469	2.747
GLY-723	-0.414	PHE-470	-1.623
GLY-724	-0.694	PHE-471	-0.08
VAL-725	-0.203	ALA-472	-0.142
ASP-741	2.816	LEU-473	-1.53
TRP-742	-0.314	ILE-474	-0.076
ASN-743	-1.56	ASN-668	0.265
PRO-744	-6.692	SER-719	-0.013
ALA-745	-7.402	SER-720	0.007
THR-746	-3.942	LYS-721	-0.218
ASP-747	3.342	GLY-723	-0.034
ILE-748	-3.724	GLY-724	-0.057
GLN-749	0.721	ASP-741	0.816
ALA-750	-0.415	TRP-742	-0.189
GLN-782	-0.586	ASN-743	-0.437
LYS-785	-1.811	PRO-744	-0.246
GLN-786	-1.917	ALA-745	-0.946
GLY-787	-0.271	THR-746	-0.558
LEU-788	-0.535	ASP-747	0.146
CYS-789	-1.88	GLN-749	0.378
GLY-790	-0.091	ALA-750	-0.025
THR-798	-2.575	LYS-785	0.292
SER-799	-0.544	CYS-789	-0.047
GLU-800	-0.12	GLY-790	-0.009
		VAL-793	-0.048
		THR-798	-0.136
		SER-799	-0.242
		GLU-800	0.562

#### Steered molecular dynamics (SMD)

**Fig. 6:**
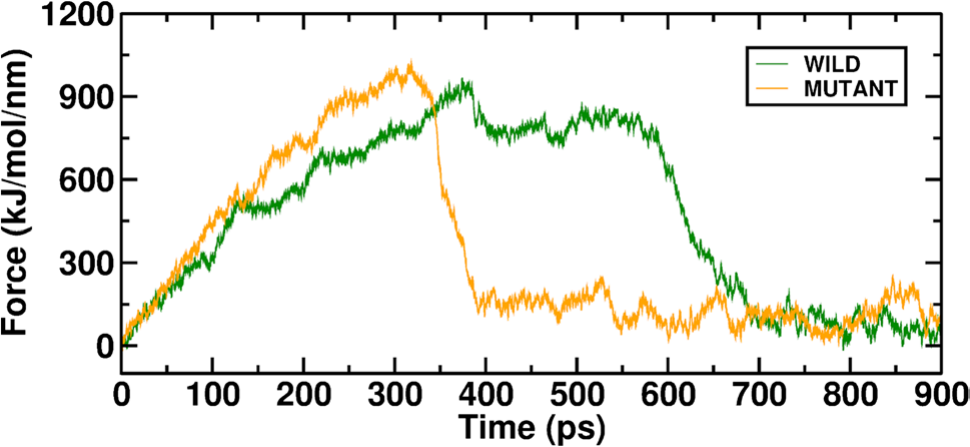
In MD simulation, the SMD force averaged over the Wild-ATP (RAD54B) and Mutant-ATP complexes

On-time scales attainable by molecular dynamics simulations steered molecular dynamics (SMD) causes ligands to unbind from their biomolecules and change in conformation. A system is subjected to time-dependent external influences, and the system's responses are studied. In the present work, SMD simulations pulled ATP along its unbinding path. SMD simulations were run using the stiff spring constant to pull the ATP molecule from its binding site on the RAD54B protein. In the case of the Wild-ATP complex, the force value increased along the time evolution of the pulling simulation during the initial unbinding phase of ATP (0–600 ps). ATP was released from the binding pocket with a peak rupture force of 925.27 kJ mol^−1^ nm^−1^. However, in the Mutant-ATP complex, the ATP molecule was found to be released from the ATP binding pocket with a similar peak rupture force value of 978.37 kJ mol^−1^ nm^−1^. The forces start to decline after reaching a maximum, indicating disruption of strong non-bonded interactions between ATP and the lining residues of the ATP binding pocket. The force profile of the Wild-ATP complex was relatively flat after 700 ps, indicating only modest interaction in the dissociation path. However, the force plot of the Mutant-ATP complex became flat after 400 ps. Thus, the ATP molecule is difficult to pull out of the binding pocket in the case of wild protein, as strong non-bonded interactions exist between ATP and the pocket-lining residues. This fact is reflected in the force plot of the Wild-ATP complex, where a force of 900 kJ mol^−1^ nm^−1^ was applied in large timeframes (350-600 ps) to rupture all interactions and release the molecule. This was not observed in the case of the Mutant-ATP complex as the pull force was able to rupture the interactions between ATP and the protein in a very short time frame (300-340 ps), indicating that the ATP molecule weakly bound in the mutant protein, thereby capable of being extracted very quickly from the complex. The Wild-ATP and Mutant-ATP complexes of the SMD graph are illustrated in (Fig. 6).

**Fig. 7:**
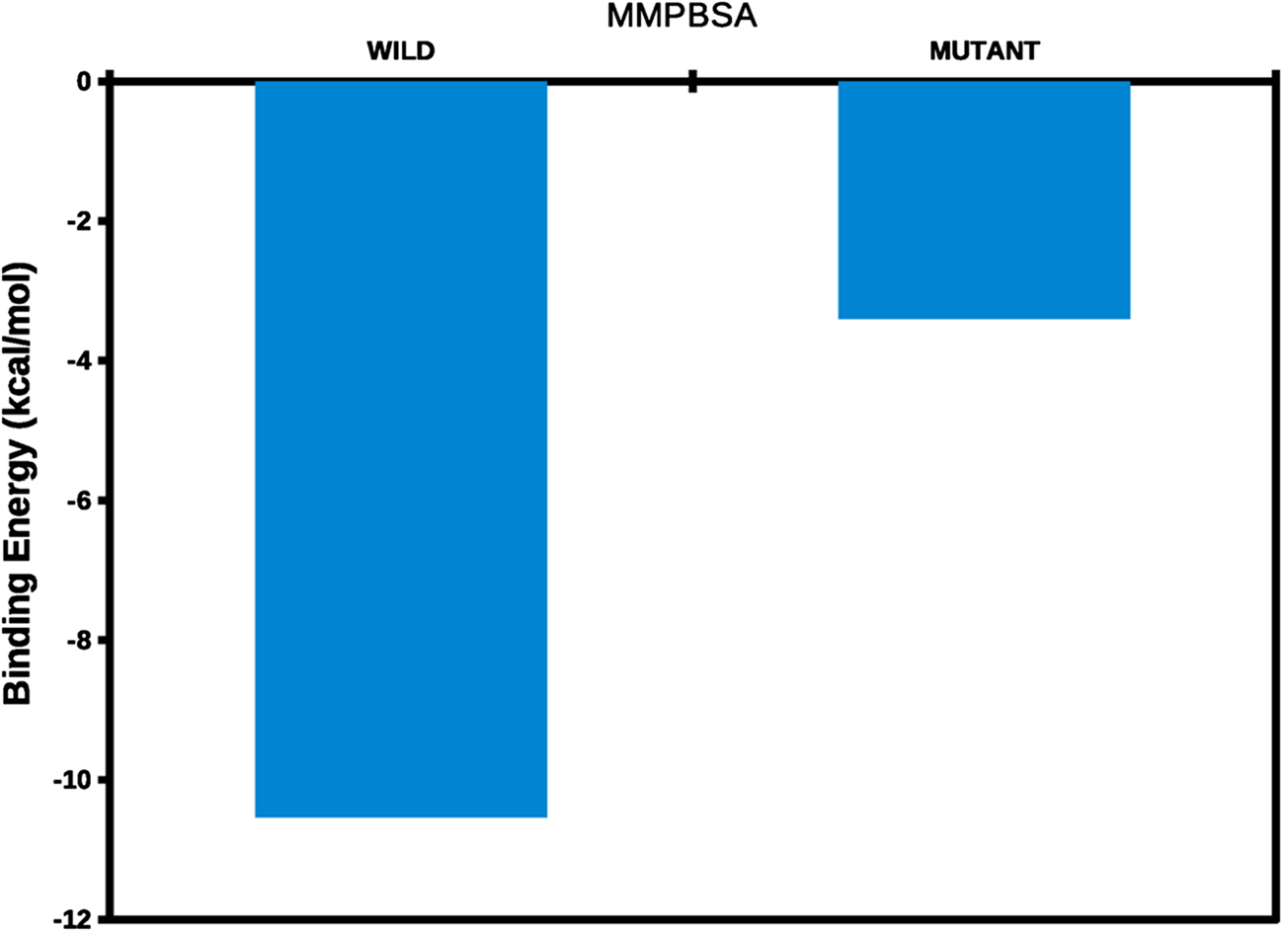
Binding free energy of Wild-ATP (RAD54B) and Mutant-ATP complexes computed in the active site pocket of the time-averaged structure of both protein types

Wild-ATP and mutant-ATP complexes' binding affinities were measured. Within the active site, we looked at the differential binding capacity. (Fig. 6) compares the binding strength of Wild-ATP and Mutant-ATP complexes examined via the MM-PBSA method. We determined residue-level contributions to the interaction energy throughout a steady simulation trajectory.

(Fig. 7) demonstrates that the binding energy of Wild-ATP in the active center pocket was found to be -10.5416 kcal/mol, while the Mutant-ATP procured binding energy of -3.401 kcal/mol. The MM-PBSA suggested that Wild-ATP exhibited significant binding energy in the active binding pocket. They show that Wild-ATP interacted with the active site pocket more favourably than Mutant-ATP (Table 5). Hence, the above computational analyses exhibited the importance of wild RAD54B compared to the mutant RAD54B. These differences would make the mutant RAD54B either disrupt its functional role due to mutation or could influence the pathway that the RAD54B involved. However, a wider population and biochemical techniques are required to validate the RAD54B mutations in AD patients.

## Conclusion

Identifying genetic modifiers is crucial in understanding their significant contribution to the disease's pathogenesis. In the current study, 37 whole exome sequences of LOAD samples were investigated to identify rare variants specific to the East-Asian population. Around 17 genes with potentially deleterious variants not previously studied in LOAD cases were identified. These rare variants are reported for the first time in individuals with LOAD from our study. Association signals that GWAS previously discovered with common and primarily non-functional variants cannot be explained by rare variants. However, our study findings explicitly showed the structural mechanisms of RAD54B mutation and also, more target-based biological experiments should be implemented to learn more about how these genes contribute to AD pathogenesis. Nevertheless, the identified variants were not previously linked to AD, highlighting the capacity of the whole-exome sequencing method to find uncommon variations linked to AD. These rare variants could be considered novel predisposing genetic factors for LOAD and might increase neuro-degeneration.

## Data Availability

Data sharing is not applicable- no new data is generated, or the article describes entirely theoretical research.
